# Modified Tal (M-Tal) Score as a Predictor of Outcomes in Infants with Bronchiolitis: A Prospective Study

**DOI:** 10.3390/pediatric18030069

**Published:** 2026-05-17

**Authors:** Ipshita Magh, Rashmi Ranjan Das, Ritwick Mohapatra, Swarupa Panda, Nirmal Kumar Mohakud

**Affiliations:** 1 Department of Pediatrics, SCB Medical College & Hospital, Cuttack 753007, Odisha, India; ipshita.magh@gmail.com (I.M.); pandadrswarupa@gmail.com (S.P.); 2 Department of Pediatrics, All India Institute of Medical Sciences, Bhubaneswar 751019, Odisha, India; 3 Department of Pediatrics, Kalinga Institute of Medical Sciences, Kalinga Institute of Industrial Technology Deemed to be University, Bhubaneswar 751024, Odisha, India

**Keywords:** bronchiolitis, modified Tal score, lower respiratory tract infection, respiratory support, severity score, observational study

## Abstract

Background/Objectives: Bronchiolitis is the leading cause of hospitalization among infants, and early prediction of disease severity and clinical outcomes remains challenging. The Modified Tal (M-Tal) score is a clinical tool used to assess severity; however, its utility in predicting outcomes requires further validation. Methods: This prospective study was conducted over a 2-year period at a tertiary care teaching hospital. Infants aged 1–12 months diagnosed with moderate-to-severe bronchiolitis were enrolled. Demographic, clinical, management, and outcome data were recorded. Results: A total of 120 infants were included (mean age 7.7 months; 61.7% males). Moderate bronchiolitis accounted for 81.7% of cases. The mean duration of illness at admission was 4.1 days. Severe bronchiolitis was associated with significantly longer duration of oxygen therapy (*p* = 0.034) and hospital stay (*p* = 0.028). Each one-point increase in M-Tal score was associated with an increase of 0.69 days in hospital stay (*p* = 0.012), 9.8 h in oxygen requirement (*p* < 0.01), and 0.32 days in PICU stay (*p* = 0.04). Conclusions: The M-Tal score at admission is a useful predictor of clinical outcomes in infants with moderate-to-severe bronchiolitis. Higher scores are associated with increased need for respiratory support and prolonged hospitalization, supporting its role in early risk stratification and management planning.

## 1. Introduction

Bronchiolitis is an acute viral lower respiratory tract infection that primarily affects infants and young children, with peak incidence in the first year of life [1]. It remains the most common cause of hospitalization in infants worldwide and contributes substantially to pediatric morbidity, particularly in low- and middle-income countries (LMICs) [2]. Respiratory syncytial virus (RSV) is the most frequent etiological agent, although other viruses such as rhinovirus, human metapneumovirus, and parainfluenza also contribute [3].

Despite its high prevalence, predicting disease severity and clinical trajectory remains difficult [4]. Most infants experience mild, self-limiting illness; however, a subset develops severe disease requiring oxygen supplementation, intensive care, or mechanical ventilation. Early identification of infants at risk for severe outcomes is crucial for appropriate triage, resource allocation, and management [1,2,3,4].

To address this gap, several clinical severity scoring systems have been developed, including the Respiratory Distress Assessment Instrument (RDAI), Wang score, and Tal score [5,6,7,8]. The Modified Tal (M-Tal) score is adapted from the original Tal score and is a simple, bedside clinical scoring system that incorporates four key clinical parameters: respiratory rate, degree of wheezing, use of accessory muscles (retractions), and oxygen saturation [8]. Each parameter is graded on a standardized scale, and the total score reflects overall disease severity, typically categorized as mild (a score of ≤ 5), moderate (a score of 6–10), or severe (a score of ≥ 11).

Despite its conceptual advantages, evidence regarding the predictive validity of the M-Tal score for clinically meaningful outcomes remains limited, particularly in LMIC settings. Existing studies suggest an association between higher scores and increased severity; however, robust prospective data evaluating its relationship with outcomes such as duration of hospitalization, oxygen requirement, need for intensive care, and mechanical ventilation are scarce [8,9]. Furthermore, healthcare systems in resource-limited settings require simple, cost-effective tools for risk stratification that can guide early triage and optimize the allocation of limited critical care resources. In such contexts, a validated clinical scoring system like the M-Tal score could play a crucial role in improving patient outcomes and healthcare efficiency.

Therefore, this prospective study was conducted to evaluate the association between admission M-Tal score and clinical outcomes in infants with moderate-to-severe bronchiolitis admitted to a tertiary care center. Continuous outcomes included duration of hospital stay, duration of oxygen therapy, and duration of PICU stay, while binary outcomes included PICU admission and requirement of mechanical ventilation. We hypothesized that higher M-Tal scores at admission would be independently associated with worse clinical outcomes.

## 2. Materials and Methods

### 2.1. Study Design

This prospective observational study was conducted in the Department of Pediatrics of a tertiary care teaching hospital in Eastern India, with a dedicated pediatric facility of approximately 200 beds. The study was conducted from July 2023 to June 2025, encompassing two peak bronchiolitis seasons; however, participant recruitment and data collection commenced only after ethics approval was obtained on 23 November 2023 from the Institute Ethics Committee. Written informed consent was obtained from parents or caregivers.

### 2.2. Study Population

Infants aged 1 to 12 months diagnosed with bronchiolitis were eligible for inclusion. Bronchiolitis was defined clinically based on the presence of a first episode of wheezing associated with signs of viral upper respiratory infection. Only infants with moderate-to-severe bronchiolitis (M-Tal score ≥ 6) were included because the study aimed to evaluate clinically meaningful outcomes among hospitalized patients requiring active respiratory management. Infants were excluded if they had congenital heart disease, chronic lung disease (e.g., bronchopulmonary dysplasia), immunodeficiency disorders, genetic syndromes, or recent hospitalization for respiratory illness (within 2 weeks). Prematurity was not an exclusion criterion.

### 2.3. Data Collection

Data were collected using a standardized case record form (CRF). The following data were recorded: (a) demographic variables—age (months) and sex; (b) clinical variables—duration of illness prior to admission, vital signs at admission, M-Tal score at admission, and severity classification (moderate: 6–10; severe: ≥11); and (c) outcomes. The previously validated original M-Tal score was used without modification or translation [8]. The details of the M-Tal score and its interpretations are given in Supplementary Table S1.

Outcomes measured were as follows: (a) primary outcomes—duration of hospital stay and duration of oxygen therapy; (b) secondary outcomes—need for advanced respiratory support (both invasive and non-invasive), pediatric intensive care unit (PICU) admission, and duration of PICU stay.

Decisions regarding the escalation of respiratory support were based on the attending physician’s clinical judgment, following institutional protocols. While specific, rigid quantitative thresholds (e.g., specific blood gas values or exact FiO_2_ cut-offs) were not universally standardized for all patients, escalation to HHHFNC was typically initiated upon clinical evidence of persistent or worsening respiratory distress (i.e., increased work of breathing, accessory muscle use, grunting, or nasal flaring); failure to maintain SpO_2_ > 92% despite low-flow oxygen support; or clinical indicators of impending respiratory failure. PICU admission was indicated for severe respiratory distress, apnea, impending respiratory failure, or the need for advanced respiratory support. Mechanical ventilation was initiated in cases of worsening respiratory failure, severe hypoxemia, hypercapnia, recurrent apnea, or exhaustion, according to institutional protocols.

### 2.4. Sample Size Calculation

The sample size was calculated to detect a significant association between the M-Tal score and duration of hospital stay, assuming a linear relationship between severity score and outcome. Based on prior studies evaluating clinical severity scores in bronchiolitis, a moderate effect size (correlation coefficient, r ≈ 0.25) between severity score and clinical outcomes was anticipated [8,9]. Using a two-tailed alpha of 0.05 and a power of 80%, the minimum required sample size to detect this correlation was calculated to be 120 subjects.

The sample size calculation was primarily based on detecting an association between M-Tal score and continuous outcomes. Although the study allowed exploratory multivariable regression analyses, the relatively small number of adverse events, particularly mechanical ventilation, may limit statistical power for some logistic regression models.

### 2.5. Statistical Analysis

The data were entered into a Microsoft Excel spreadsheet. IBM SPSS Statistics for Windows version 21 (IBM Corporation, Armonk, NY, USA) was used for analysis. Continuous variables were assessed for normality using histogram inspection and the Shapiro–Wilk test. Comparisons between moderate and severe bronchiolitis groups were performed using the independent *t*-test or Mann–Whitney U test for continuous variables and chi-square or Fisher’s exact test for categorical variables, as appropriate. The association between M-Tal score and continuous outcomes (duration of hospital stay, oxygen therapy, and PICU stay) was assessed using linear regression analysis. Variables with *p* < 0.1 in univariate analysis and clinically relevant covariates (age, sex, and duration of illness prior to admission) were included in multivariate linear regression models. Multicollinearity among independent variables was assessed using the variance inflation factor (VIF), with a VIF < 2 indicating low collinearity [10]. For binary outcomes (PICU admission and requirement of mechanical ventilation), multivariate logistic regression analysis was performed to estimate adjusted odds ratios (aORs) with 95% confidence intervals (CIs). Model performance was evaluated using Nagelkerke R^2^ to assess explained variance and the Hosmer–Lemeshow goodness-of-fit test [11]. Regression assumptions, including linearity, homoscedasticity, and residual distribution, were evaluated. All statistical tests were two-tailed, and a *p*-value < 0.05 was considered statistically significant.

## 3. Results

### 3.1. Baseline Characteristics

**Figure 1:**
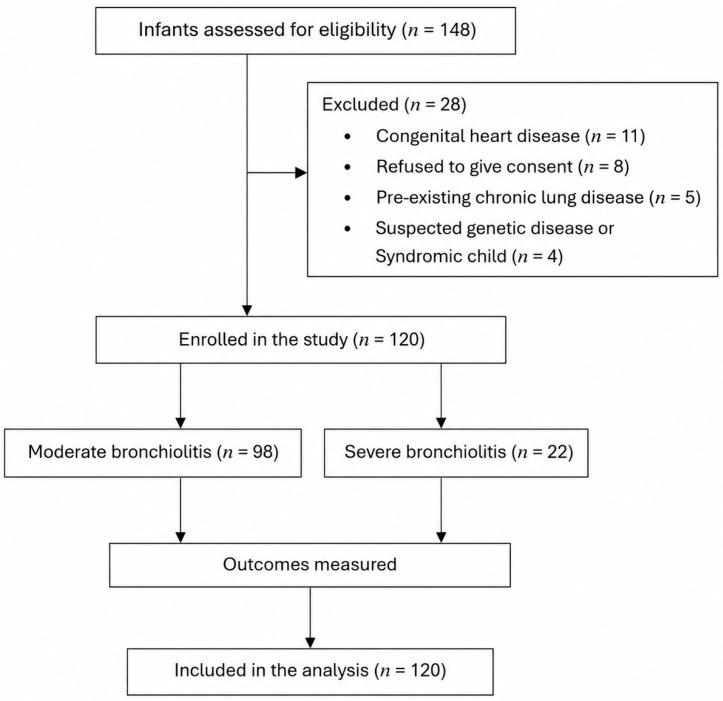
Flow of study participants.

A total of 120 infants with moderate-to-severe bronchiolitis were enrolled during the study period. The flow of participants is illustrated in Figure 1.

The baseline demographic and clinical characteristics of the study population are summarized in Table 1.

The mean age of the enrolled infants was 7.7 ± 2.8 months. Male infants comprised 61.7% of the study population, and 37 infants (30.8%) were born preterm. The mean duration of illness prior to hospital admission was 4.1 ± 1.6 days, indicating that most infants presented within the first week of illness. Based on the Modified Tal (M-Tal) score at admission, the majority were classified as having moderate bronchiolitis (81.7%). This distribution reflects the clinical profile of hospitalized bronchiolitis, where moderate cases constitute the bulk, but a significant minority presents with severe illness requiring closer monitoring and advanced care.

### 3.2. Respiratory Support and Clinical Course

All infants required some form of respiratory support during hospitalization, the most common being low-flow oxygen via nasal cannula (65%). A substantial proportion (31.7%) required escalation to a heated humidified high-flow nasal cannula (HHHFNC), indicating moderate-to-severe respiratory compromise. Mechanical ventilation was required in 10.8% of cases, reflecting the subset of patients with critical illness. Notably, infants in the severe bronchiolitis group had a significantly higher requirement for advanced respiratory support modalities, particularly HHHFNC and invasive ventilation, compared to those in the moderate group.

### 3.3. Comparison of Outcomes Between Moderate and Severe Bronchiolitis

A comparison of clinical outcomes between infants with moderate and severe bronchiolitis revealed significant differences across all major outcome measures (Table 2). Infants with severe bronchiolitis had a longer duration of oxygen therapy and hospital stay compared to those in the moderate group. The need for PICU admission and the duration of PICU stay were significantly higher in the severe group compared to the moderate group. The requirement for mechanical ventilation was also substantially higher among infants with severe disease, underscoring the increased severity and resource utilization in this subgroup.

### 3.4. Association Between M-Tal Score and Clinical Outcomes

To further evaluate the relationship between M-Tal score and clinical outcomes, regression analyses were performed. On univariate linear regression analysis, increasing M-Tal score demonstrated a significant positive association with all continuous outcome variables. Specifically, each one-point increase in M-Tal score was associated with a 0.69-day increase in hospital stay (95% CI: 0.15–1.23, *p* = 0.012), a 9.8 h increase in duration of oxygen therapy (95% CI: 4.2–15.4, *p* < 0.001), and a 0.32-day increase in PICU stay (95% CI: 0.02–0.62, *p* = 0.04) (Table 3).

**Figure 2:**
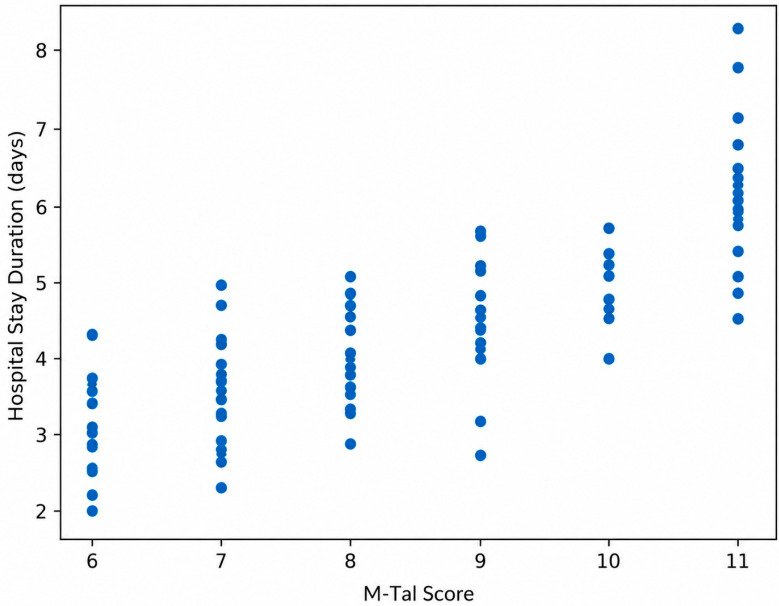
Scatter plot showing a linear relationship between M-Tal score and duration of hospital stays.

A positive linear relationship between M-Tal score and duration of hospital stay is illustrated in Figure 2. These findings indicate a positive linear association between clinical severity at admission and subsequent healthcare utilization.

### 3.5. Multivariate Linear Regression Analysis of Factors Associated with Clinical Outcomes

To account for potential confounding factors, multivariate linear regression analysis was performed, adjusting for age, sex, and duration of illness prior to admission. The M-Tal score remained an independent and statistically significant predictor of all primary and secondary continuous outcomes. Each unit increase in M-Tal score was associated with a 0.62-day increase in hospital stay (*p* = 0.006), an 8.9 h increase in oxygen requirement (*p* < 0.001), and a 0.28-day increase in PICU stay (*p* = 0.022).

In addition to the M-Tal score, younger age and prematurity were independently associated with worse outcomes (Table 4).

The multivariate linear regression models demonstrated acceptable explanatory performance. The adjusted R^2^ values were 0.31 for duration of hospital stay, 0.38 for duration of oxygen therapy, and 0.22 for duration of PICU stay, indicating that the included variables explained a moderate proportion of the variability in clinical outcomes.

### 3.6. Multivariate Logistic Regression Analysis of Factors Associated with Adverse Outcomes

The association between M-Tal score and adverse binary outcomes was further evaluated using multivariate logistic regression analysis. The results demonstrated that higher M-Tal scores were strongly associated with increased odds of both PICU admission and mechanical ventilation. Specifically, each unit increase in M-Tal score increased the odds of PICU admission by 42% (adjusted odds ratio [aOR] 1.42, 95% CI: 1.12–1.81, *p* = 0.004). Similarly, the odds of requiring mechanical ventilation increased by 67% with each unit increase in M-Tal score (aOR 1.67, 95% CI: 1.21–2.29, *p* = 0.002). The relatively small number of mechanical ventilation events may have resulted in model overfitting and unstable estimates; therefore, these findings should be interpreted cautiously and considered exploratory.

Other factors, including younger age, prematurity, and longer duration of illness prior to admission, were also independently associated with an increased risk of adverse outcomes in logistic regression models. The models demonstrated good fit and acceptable predictive performance, as indicated by Nagelkerke R^2^ values of 0.34 for PICU admission and 0.39 for mechanical ventilation, along with non-significant Hosmer–Lemeshow test results (*p* > 0.05) (Table 5).

## 4. Discussion

In this prospective study of infants with moderate-to-severe bronchiolitis, we found that the Modified Tal (M-Tal) score at admission is a robust and independent predictor of clinically important outcomes, including duration of hospital stay, oxygen therapy, pediatric intensive care unit (PICU) admission, and need for mechanical ventilation. These findings reinforce the clinical utility of a simple bedside scoring system for early risk stratification, particularly in resource-constrained healthcare settings.

A key strength of our study is the demonstration of a graded, dose–response relationship between M-Tal score and clinical outcomes, particularly duration of hospitalization. Each incremental increase in M-Tal score was associated with a significant prolongation of hospital stay, even after adjustment for confounders. This finding is consistent with prior studies evaluating clinical severity scores in bronchiolitis, which have reported similar associations with length of stay and healthcare utilization [6,7,8,9,12,13]. However, most previous studies were retrospective or cross-sectional; our prospective design adds methodological strength and supports the temporal relationship between admission severity and subsequent outcomes.

The association between M-Tal score and duration of oxygen therapy further highlights its clinical relevance. Oxygen requirement is a central determinant of disease severity and hospitalization in bronchiolitis, and it often drives decisions regarding admission and discharge [14,15]. Our finding that higher M-Tal scores independently predict prolonged oxygen therapy is consistent with previous literature demonstrating correlations between clinical severity scores and hypoxemia [8,9]. This reinforces the potential role of M-Tal score in anticipating respiratory support needs early in the disease course.

Various factors affect the severity of bronchiolitis and the need for PICU admission and mechanical ventilation [16,17]. Importantly, our study extends existing evidence by demonstrating that the M-Tal score is also a strong predictor of critical care outcomes, including PICU admission and mechanical ventilation. Each unit increase in M-Tal score significantly increased the odds of PICU admission (aOR 1.42) and invasive ventilation (aOR 1.67). These findings are clinically significant, as early identification of infants at high risk for deterioration can facilitate timely escalation of care, optimize monitoring, and potentially improve outcomes. Similar associations have been described in studies evaluating early warning scores and bronchiolitis severity indices, although the magnitude of effect observed in our study underscores the predictive strength of the M-Tal score [6,7,8,9].

Several clinical scoring systems have been developed for bronchiolitis, including the Respiratory Distress Assessment Instrument (RDAI), Wang score, and original Tal score [5,6,7]. The RDAI focuses primarily on wheezing and retractions, whereas the Wang score incorporates respiratory rate, wheezing, retractions, and general condition. While these tools are widely used in research settings, their clinical applicability is sometimes limited by complexity or a lack of integration of oxygen saturation. The M-Tal score offers several advantages over these systems. First, it incorporates oxygen saturation, a critical parameter in bronchiolitis management, which is not included in all traditional scores. Second, it is simple and rapid to administer at the bedside, making it more feasible for routine clinical use, especially in busy or resource-limited settings. Third, prior validation studies have demonstrated its reliability and predictive value for disease severity [8,9]. Our study builds upon this evidence by providing prospective data demonstrating its association with both continuous and categorical outcomes, including critical care utilization. Despite these advantages, direct head-to-head comparisons between M-Tal and other scoring systems remain limited. Future studies should focus on comparative validation to determine the most accurate and practical tool for clinical use.

In addition to the M-Tal score, we identified younger age as an independent predictor of adverse outcomes, including prolonged hospitalization and oxygen requirement. This finding is well supported by existing literature, which consistently identifies young age as a major risk factor for severe bronchiolitis [16,17,18,19,20]. Physiological factors such as smaller airway caliber, immature immune responses, and reduced respiratory reserve likely contribute to this increased vulnerability.

Prematurity also emerged as an important predictor of adverse outcomes in our cohort. Premature infants had longer hospitalization, increased oxygen requirement, and a higher likelihood of intensive care admission and mechanical ventilation. These findings are biologically plausible because preterm infants have immature lung architecture, reduced airway caliber, impaired mucociliary clearance, and lower respiratory reserve, all of which increase vulnerability to severe viral lower respiratory tract infections. Our findings are consistent with previous studies identifying prematurity as a major risk factor for severe bronchiolitis and critical care utilization [16,18,20].

We also observed that a longer duration of illness prior to admission was associated with worse outcomes. Previous studies have found similar associations [21,22]. This may reflect disease progression due to delayed presentation or barriers to healthcare access, particularly in LMIC settings. Early recognition and timely referral of infants with bronchiolitis are therefore critical to improving outcomes. These findings highlight the importance of integrating clinical scoring systems with broader public health strategies aimed at improving access to care.

Interestingly, although male infants were more frequently affected, gender was not independently associated with disease severity or outcomes after adjustment. This suggests that while male sex may be associated with increased susceptibility to bronchiolitis, it does not significantly influence clinical progression once the disease is established, consistent with prior studies [8,9,12,20]. However, some studies have found a significant association between disease severity and male gender [19,23,24,25].

The findings of this study have important implications for clinical practice. The M-Tal score is a simple, non-invasive, and cost-effective tool that can be easily incorporated into routine clinical assessment. Its ability to predict both intermediate outcomes (oxygen requirement, hospital stay) and critical outcomes (PICU admission, mechanical ventilation) makes it particularly valuable for early triage and decision-making. From a clinical perspective, even a one-point increase in M-Tal score translates into substantially longer hospitalization and oxygen requirement, indicating greater healthcare utilization and resource burden. Such differences may assist clinicians in anticipating monitoring needs, counseling caregivers, and optimizing bed allocation and respiratory support planning. In resource-limited settings, where access to advanced diagnostics and intensive care may be constrained, the use of such a scoring system can facilitate prioritization of high-risk patients, optimize resource allocation, and potentially improve patient outcomes.

The strengths of our study include its prospective design, which allows for systematic and real-time data collection, and the use of a validated clinical scoring system. We evaluated a comprehensive set of outcomes and employed robust statistical methods, including multivariate regression analysis, to account for confounding factors. The consistency of findings across multiple analytical approaches strengthens the validity of our conclusions. However, several limitations should be considered. This was a single-center study, which may limit generalizability. We did not perform viral etiological testing, and therefore, the influence of specific pathogens could not be assessed. It has been observed that different viruses, as well as viral co-infections, affect the outcomes of bronchiolitis [26,27,28,29]. Additionally, the interobserver reliability of M-Tal scoring was not formally assessed. Variability in clinical assessment between observers may affect reproducibility and external validity across different healthcare settings. Although prematurity was included in adjusted analyses, residual confounding from other unmeasured variables, such as birth weight, nutritional status, feeding difficulty, environmental exposures, and viral etiology, may still have influenced outcomes.

Also, we did not employ rigid, predefined quantitative thresholds for all parameters, such as specific partial pressure of oxygen (PaO_2_) or carbon dioxide (PaCO_2_) values, or fixed FiO_2_ increments for escalation; there is inherent variability in how clinical decision-making was applied across patients. The number of mechanical ventilation events was relatively small, which may limit the precision and stability of logistic regression estimates. Finally, the study included only infants with moderate-to-severe bronchiolitis, and the findings cannot be extrapolated to infants with mild disease managed in outpatient settings.

Future research should focus on multicenter validation studies adopting standardized, protocol-driven criteria for escalation to minimize such variability across diverse healthcare settings to confirm the generalizability of our findings. Comparative studies evaluating the performance of the M-Tal score against other severity scores are also needed. Additionally, integrating clinical scoring systems with biomarkers or predictive algorithms may further enhance risk stratification [30].

## 5. Conclusions

Although further multicenter validation studies are needed, the M-Tal score appears to be a useful bedside tool for early risk stratification in hospitalized infants with moderate-to-severe bronchiolitis. Higher scores at admission are associated with increased respiratory support requirements, longer duration of oxygen therapy, prolonged hospitalization, and increased PICU stay.

## Figures and Tables

**Table 1:** Baseline characteristics and clinical profile of study population (*n* = 120).

Variables	Overall (*n* = 120)
Age (months), mean ± SD	7.7 ± 2.8
Age group, *n* (%) • 1–3 months • 3–10 months • >10 months	18 (15.0) 85 (70.8) 17 (14.2)
Male gender, *n* (%)	74 (61.7)
Preterm infants, *n* (%)	37 (30.8)
Duration of illness before admission (days), mean ± SD	4.1 ± 1.6
Severity (M-Tal score), *n* (%) • Moderate (6–10) • Severe (≥11)	98 (81.7) 22 (18.3)
Respiratory support, *n* (%) • Nasal cannula • HHHFNC • Mechanical ventilation	78 (65.0) 38 (31.7) 13 (10.8)

**Table 2:** Comparison of outcomes between moderate and severe bronchiolitis.

Outcomes	Moderate (*n* = 98)	Severe (*n* = 22)	*p* Value
Duration of oxygen therapy (days), mean ± SD	2.8 ± 1.4	3.9 ± 1.8	0.034 *
Duration of hospital stay (days), mean ± SD	4.5 ± 1.9	6.1 ± 2.3	0.028 *
PICU admission, *n* (%)	12 (12.2)	10 (45.5)	<0.001 *
Duration of PICU stay (days), mean ± SD	1.2 ± 0.8	2.4 ± 1.1	0.041 *
Mechanical ventilation, *n* (%)	5 (5.1)	8 (36.4)	<0.001 *

* *p*-value significant (< 0.05).

**Table 3:** Linear regression analysis of M-Tal scores and clinical outcomes.

Outcome Variables	β Coefficient (95% CI)	*p* Value
Duration of hospital stay (days)	0.69 (0.15–1.23)	0.012 *
Duration of oxygen therapy (hours)	9.8 (4.2–15.4)	<0.001 *
Duration of PICU stay (days)	0.32 (0.02–0.62)	0.040 *

* *p*-value significant (< 0.05).

**Table 4:** Multivariate linear regression analysis of factors affecting clinical outcomes.

Variables	Adjusted β Coefficient (95% CI)	*p* Value
Duration of hospital stay (days)		
M-TAL score (per unit increase)	0.62 (0.18, 1.06)	0.006 *
Age (months)	−0.08 (−0.15, −0.01)	0.024 *
Male gender	0.21 (−0.64, 1.06)	0.62
Duration of illness before admission (days)	0.34 (0.08, 0.60)	0.011 *
Prematurity	0.74 (0.12, 1.36)	0.02 *
Duration of oxygen therapy (hours)		
M-TAL score (per unit increase)	8.9 (3.7, 14.1)	<0.001 *
Age (months)	−1.2 (−2.3, −0.1)	0.032 *
Male gender	2.6 (−7.4, 12.6)	0.61
Duration of illness before admission (days)	5.1 (1.4, 8.8)	0.008 *
Prematurity	7.2 (1.1, 13.3)	0.021 *
Duration of PICU stay (days)		
M-TAL score (per unit increase)	0.28 (0.04, 0.52)	0.022 *
Age (months)	−0.03 (−0.07, 0.1)	0.14
Male gender	0.18 (−0.42, 0.78)	0.55
Duration of illness before admission (days)	0.16 (0.02, 0.3)	0.026 *
Prematurity	0.21 (−0.04, 0.46)	0.098

* *p*-value significant (<0.05).

**Table 5:** Multivariate logistic regression analysis of factors affecting adverse outcomes.

Variables	Adjusted Odds Ratio (aOR)	95% CI	*p* Value
PICU admission			
M-Tal score (per unit increase)	1.42	1.12, 1.81	0.004 *
Age (per month increase)	0.91	0.83, 0.99	0.031 *
Male gender	1.18	0.52, 2.68	0.69
Duration of illness before admission (per day)	1.29	1.05, 1.58	0.015 *
Prematurity	1.96	1.08, 3.58	0.027 *
Mechanical ventilation			
M-Tal score (per unit increase)	1.67	1.21, 2.29	0.002 *
Age (per month increase)	0.88	0.78, 0.99	0.037 *
Male gender	1.26	0.46, 3.44	0.65
Duration of illness before admission (per day)	1.34	1.06, 1.7	0.013 *
Prematurity	2.31	1.14, 4.68	0.019 *

* *p*-value significant (<0.05).

## Data Availability

The data presented in this study are available upon request from the corresponding author. The data are not publicly available due to privacy restrictions.

## Supplementary Materials

The following supporting information can be downloaded at: https://www.mdpi.com/article/10.3390/pediatric18030069/s1, Table S1: Components and interpretation of the Modified Tal (M-Tal) score.

## Abbreviations

The following abbreviations are used in this manuscript:

M-Tal score	Modified Tal score
HHHFNC	Heated humidified high-flow nasal cannula
PICU	Pediatric intensive care unit
LMICs	Low- and middle-income countries
RSV	Respiratory syncytial virus
RDAI	Respiratory Distress Assessment Instrument
CRF	Case Record Form
VIF	Variance inflation factor
SD	Standard deviation
CI	Confidence interval
OR	Odds ratio
aOR	Adjusted odds ratio
