# Supplementary material for: Modified Tal (M-Tal) Score as a Predictor of Outcomes in Infants with Bronchiolitis: A Prospective Study
Source: Pediatr Rep. 2026 May 17;18(3):69. doi: 10.3390/pediatric18030069 (PMC13214627; doi:10.3390/pediatric18030069)
Supplement: Supplementary file 1 [file pediatrrep-18-00069-s001.zip › pediatrrep-4302227-supplementary.pdf]

Supplementary Table S1: Components and Interpretation of the Modified Tal (M-Tal) Score

Clinical parameter	Score 0	Score 1	Score 2	Score 3
Respiratory rate (breaths/minute)	<30	31–45	46–60	>60
Wheezing	None	Terminal expiratory wheeze only	Entire expiration audible	Inspiratory and expiratory wheeze audible without stethoscope / markedly diminished air entry
Retractions (use of accessory muscles)	None	Mild intercostal retractions	Moderate intercostal and substernal retractions	Severe generalized retractions with nasal flaring
Oxygen saturation in room air (%)	>95	92–95	89–91	<89

Interpretation of M-Tal score

Total score	Severity classification
≤5	Mild bronchiolitis
6–10	Moderate bronchiolitis
≥11	Severe bronchiolitis

Footnote: The M-Tal score used in the present study was identical to the previously validated Modified Tal score described by Ilan Golan-Tripto *et al* [8]. No local modifications were applied.
